# Novel ternary nanocomposites of MWCNTs/PANI/MoS_2_: preparation, characterization and enhanced electrochemical capacitance

**DOI:** 10.1098/rsos.171365

**Published:** 2018-01-03

**Authors:** Ranran Zhang, Yu Liao, Shuangli Ye, Ziqiang Zhu, Jun Qian

**Affiliations:** School of Printing and Packaging, Wuhan University, Wuhan 430072, People's Republic of China

**Keywords:** MWCNTs, PANI, MoS_2_, nanocomposite, electrochemical capacitance

## Abstract

In this work, nanoflower-like MoS_2_ grown on the surface of multi-walled carbon nanotubes (MWCNTs)/polyaniline (PANI) nano-stem is synthesized via a facile *in situ* polymerization and hydrothermal method. Such a novel hierarchical structure commendably promotes the contact of PANI and electrolyte for faradaic energy storage. In the meanwhile, the double-layer capacitance of MoS_2_ is effectively used. The morphology and chemical composition of the as-prepared samples are characterized by scanning and transmission electron microscopies, X-ray diffraction and Fourier transform infrared spectra. The electrochemical performance of the samples is evaluated by cyclic voltammogram and galvanostatic charge–discharge measurements. It is found that the specific capacitance of the obtained MWCNTs/PANI/MoS_2_ hybrid is 542.56 F g^−1^ at a current density of 0.5 A g^−1^. Furthermore, the MWCNTs/PANI/MoS_2_ hybrid also exhibits good rate capability (62.5% capacity retention at 10 A g^−1^) and excellent cycling stability (73.71% capacitance retention) over 3000 cycles.

## 1. Introduction

In recent years, global warming and the energy crisis have accelerated the development of advanced energy storage devices. As one of the most promising candidates, supercapacitors are receiving extensive attention due to their high power/energy density, excellent cycling stability and fast charge/discharge capability [1–3]. Supercapacitors can be divided into two types depending on their energy storage mechanisms: electrical double-layer capacitors (EDLCs) and pseudo-capacitors [4–6]. For the EDLCs, various carbonaceous materials, such as carbon nanotubes (CNTs), graphene and activated carbon, have been widely employed as electrode materials [7,8]. However, the specific capacitance of carbon electrodes is relatively low. In contrast, for the pseudo-capacitor, transition metal oxides and conducting polymers (e.g. polyaniline, polypyrrole and polythiophene) are very popularly used as electrode materials with higher energy storage capacity [9,10]. Among these conducting polymers, polyaniline (PANI) has been widely used as an ideal electrode material in the construction of high-performance supercapacitors due to its high theoretical specific capacitance, good electrochemical activity, good biocompatibility, low cost and ease of fabrication [11,12]. However, the main drawback restricting the application of PANI electrodes in supercapacitors is the mechanical degradation and poor cycling stability during the charge/discharge process [13,14]. To overcome these problems, many research works have been conducted on the preparation of PANI-based composites with EDLC electrode materials for hybrid capacitors, which are conducive to enhancing the specific capacitance, mechanical stability and cycling stability [15–19].

The combination of CNTs with PANI is an effective way to enhance the specific capacitance and cycling stability of PANI. For example, Li *et al.* [20] reported three types of nanocomposites synthesized by *in situ* chemical polymerization of aniline onto double-walled carbon nanotubes (DWCNTs), single-walled carbon nanotubes (SWCNTs) and multi-walled carbon nanotubes (MWCNTs), respectively. They found that the specific capacitances were 576, 390 and 344 F g^−1^ for composites of DWCNTs/PANI, SWCNTs/PANI and MWCNTs/PANI, which were much higher than that of pure PANI (226 F g^−1^). The cycling stability of the three nanocomposites was also higher than that of pure PANI.

On the other hand, layered transition-metal dichalcogenides (WS_2_, MoS_2_ and VS_2_) have been successfully established as a new paradigm in the chemistry of nanomaterials and have aroused wide attention [21–23]. Especially, molybdenum disulfide (MoS_2_), a typical type of transition-metal dichalcogenide with a layered structure like graphene, has attracted tremendous attention, because it could be used as electrode material for supercapacitors due to its higher theoretical specific capacitance [21] and higher intrinsic fast ionic conductivity [24]. Therefore, many studies have combined PANI with MoS_2_ to yield improved electrochemical performance [25–27]. Lei *et al.* [28] reported a hierarchical core--sheath PANI@MoS_2_ nanocomposite via a hydrothermal redox reaction for high-performance electrochemical capacitor applications. They found that the nanocomposite electrode displayed a high specific capacitance of 450 F g^−1^ and excellent cycling stability (retaining 80% after 2000 charge/discharge processes), while the specific capacitance of individual PANI was 338 F g^−1^, and the PANI electrode only retained 47% of the value of the first cycle. Wang and co-workers [29] reported MoS_2_/PANI hybrid electrode material via direct intercalation of aniline monomer and doped with dodecylbenzenesulfonic acid. They found that when the loading amount of MoS_2_ was 38%, the obtained hybrid electrode exhibited a high specific capacitance of 390 F g^−1^ at a current density of 0.8 A g^−1^ and excellent cycling stability (86% retention over 1000 cycles).

The recent research works on electrochemical performance involving these materials [3,18,29–34] are listed in table 1. It can be seen that the specific capacitance of MoS_2_/PANI [29] and PANI/sulfonated MWCNTs [18] is not high and the cycling stability of CNTs-g-PANI [30] and graphene/PANI nanotube [34] is not ideal.

**Table 1.** Comparison of properties of different electrode materials.

electrode materials	specific capacitance	cycling stability	refs
MoS_2_/PANI	390 F g^−1^ at 0.8 A g^−1^	86% after 1000 cycles at 0.8 A g^−1^	[29]
CNTs-g-PANI	197.4 F g^−1^ at 1 A g^−1^	64% after 5000 cycles at 1 A g^−1^	[30]
graphene/MnO_2_/PANI	305 F g^−1^ at 1 A g^−1^	90% after 1000 cycles at 1 A g^−1^	[31]
MoS_2_/CNT	74.05 F g^−1^ at 2 A g^−1^	80.8% after 1000 cycles at 2 A g^−1^	[32]
graphene/CNT-PANI	526 F g^−1^ at 2 A g^−1^	82% after 1000 cycles at 2 A g^−1^	[3]
3D S-doped GA	445.6 F g^−1^ at 5 mV s^−1^	73.4% after 1500 cycles at 100 mV s^−1^	[33]
PANI/sMWCNTs	515.2 F g^−1^ at 20 mV s^−1^	90% after 1000 cycles at 1 A g^−1^	[18]
graphene/PANI nanotube	561 F g^−1^ at 50 mV s^−1^	61% after 500 cycles at 50 mV s^−1^	[34]
MWCNTs/PANI/MoS_2_	542.6 F g^−1^ at 0.5 A g^−1^	73.7% after 3000 cycles at 1 A g^−1^	this work

**Figure 1.**
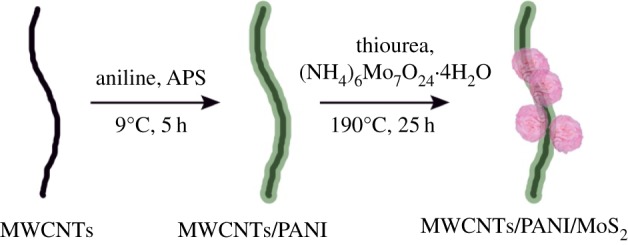
Schematic illustration for synthesis of MWCNTs/PANI/MoS_2_ ternary nanocomposites.

In this paper, we introduce both MWCNTs and MoS_2_ as a composite with PANI to be used as supercapacitor electrode material. The MWCNTs not only act as the initial supporter for the polymerization of aniline monomer, but also enhance the electrical conductivity and electrochemical properties of hybrid materials. Nanoflower-like MoS_2_ is grown on the surface of the MWCNTs/PANI nano-stem through a facile hydrothermal reaction, where MoS_2_ plays an important role in enhancing the charge storage capabilities and the cycling stability during the charge/discharge process. The preparation procedure is illustrated in figure 1. The obtained ternary nanocomposite exhibits high specific capacitance and excellent cycling stability.

## 2. Material and reagents

Thiourea was supplied by Sigma-Aldrich. MWCNTs were purchased from Chengdu Organic Chemicals Co. Ltd, Chinese Academy of Sciences. Aniline, ammonium peroxydisulfate ((NH_4_)_2_S_2_O_8_, APS) and ammonium molybdate tetrahydrate ((NH_4_)_6_Mo_7_O_24_·4H_2_O) were purchased from Wuhan Shenshi Chemical Instrument Network Co. Ltd (China). All other chemicals and solvents were used as received without further treatment.

### 2.1. Synthesis of MWCNTs/PANI nanocomposites

MWCNTs/PANI composites were synthesized by an *in situ* oxidative polymerization using the procedures in our previous work [35]. Primarily, the MWCNTs were purified and functionalized by nitric acid at 80°C for 6 h. Then, 70 mg of the purified MWCNTs was dispersed in 50 ml of 1 M HCl with ultrasonic treatment for 0.5 h. In the meantime, 150 ml of 1 M HCl solution with 0.3 g of aniline monomer was treated by stirring for 1 h at room temperature. Afterwards, the above two solutions were mixed together and sonicated for another 0.5 h. Next, 0.5 g of APS was added into the mixture and stirred for 5 h at 9°C. Finally, the mixture was filtered and washed with deionized water three times and then dried at 60°C for 12 h with vacuum. Pure PANI was prepared through the same procedure without MWCNTs.

### 2.2. Preparation of MWCNTs/PANI/MoS_2_ ternary nanocomposites

To synthesize the ternary hybrid MWCNTs/PANI/MoS_2_ nanocomposites, the as-prepared MWCNTs/ PANI (30 mg) was dispersed in 25 ml of deionized water with the help of ultrasonication for 40 min. Ammonium molybdate tetrahydrate ((NH_4_)_6_Mo_7_O_24_·4H_2_O, 0.1 g) and thiourea (0.086 g) were added into the MWCNTs/PANI suspension, and the mixture was sonicated for 10 min. Then, the obtained mixed suspension was transferred into a 50 ml Teflon-lined stainless steel autoclave and heated at 190°C for 25 h. After cooling down to room temperature naturally, the resulting black precipitates were collected by centrifugation with deionized water and then dried in vacuum at 60°C for 24 h to obtain the ternary hybrid of MWCNTs/PANI/MoS_2_, abbreviated as MPM-0.1. For comparison, ternary MWCNTs/PANIMoS_2_ nanocomposites with different amounts of ammonium molybdate tetrahydrate (0.06 g and 0.14 g) were prepared by using the same process, which were denoted as MPM-0.06 and MPM-0.14 (the mass ratio of ammonium molybdate tetrahydrate to thiourea was 1.16).

### 2.3. Preparation of the modified electrodes

The working electrode was prepared as follows: firstly, a glass carbon electrode (GCE) was prepared by polishing and ultrasonic cleaning. The obtained nanocomposites were dispersed in Nafion (1%) solution and sonicated for 1 h to form a homogeneous mixture. Then, the mixture (10 µl) was dropped onto the pretreated GCE and dried at room temperature.

The electrochemical tests (cyclic voltammetry (CV) and galvanostatic charge–discharge (GCD)) were carried out by using an Autolab (μ3AUT71018) electrochemical workstation with a three-electrode system, in which the GCE coated with the obtained samples was used as the working electrode, a platinum foil as the counter electrode and an Ag/AgCl electrode as the reference electrode in 1 M H_2_SO_4_ electrolyte. The CV curves were recorded at scan rates of 10–100 mV s^−1^ in the voltage range from −0.2 to 1 V. GCD curves were measured at different current densities of 0.5, 1, 2, 4 and 10 A g^−1^ (0–0.8 V). The specific capacitances of electrode materials were calculated according to the following equation:

2.1 $$C_{m}=I\;t/\Delta Vm,$$

where *I*, *t*, Δ*V* and *m* are the charge/discharge current (A), discharge time (s), potential change (V) and the weight of active materials (g), respectively.

## 3. Results and discussion

### 3.1. Morphology and chemical composition analysis

**Figure 2.**
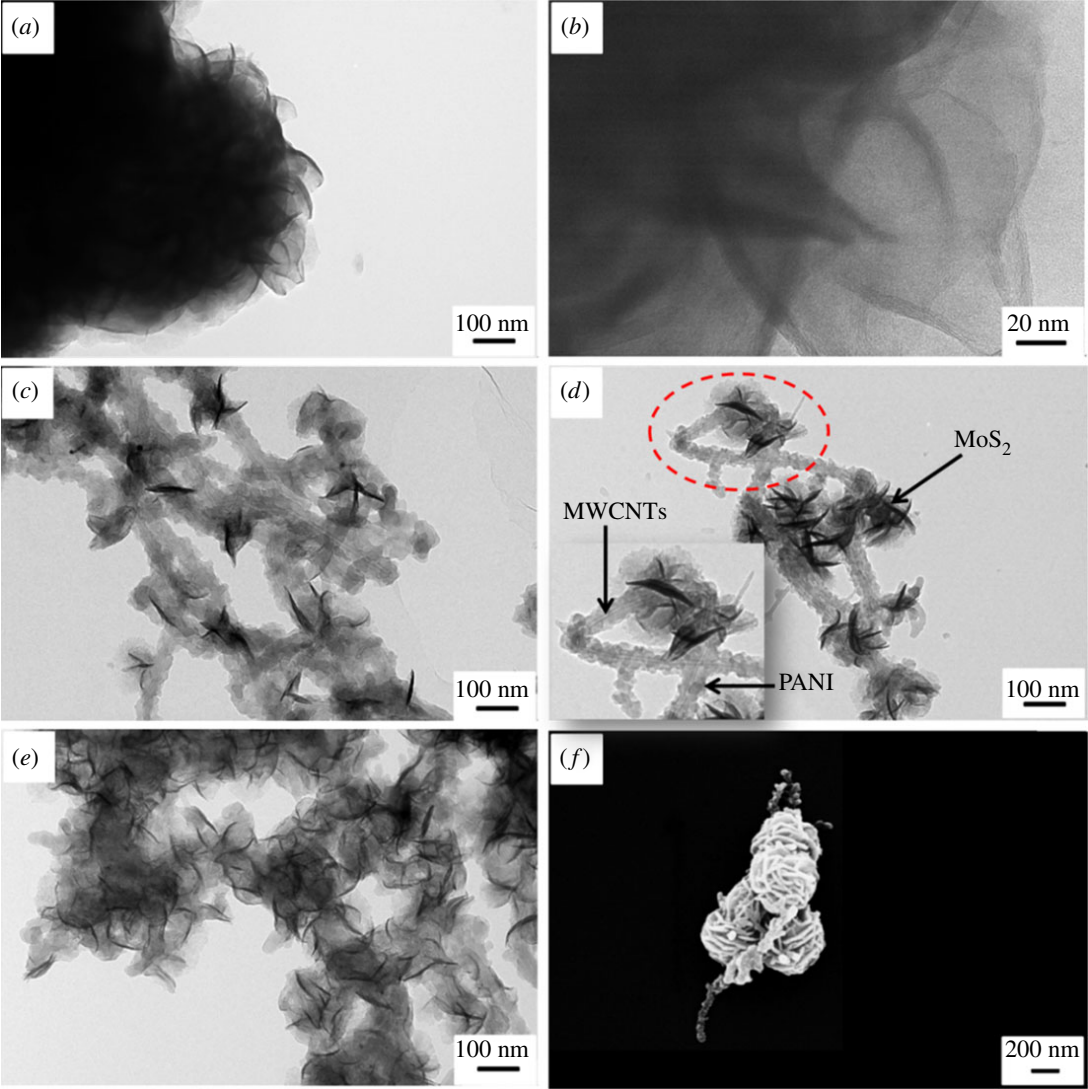
TEM images of (*a*) MoS_2_, (*b*) high magnification of MoS_2_, (*c*) MPM-0.06, (*d*) MPM-0.1, (*e*) MPM-0.14. (*f*) SEM image of MPM-0.1.

The transmission electron microscopy (TEM) and scanning electron microscopy (SEM) morphologies of the as-prepared MoS_2_ and MWCNTs/PANI/MoS_2_ nanocomposites are shown in figure 2. Figure 2*a*,*b* display the TEM images of pure MoS_2_ with different magnification. It can be seen that the layered MoS_2_ nanosheets are tightly stacked and agglomerated together. In the presence of MWCNTs/PANI acting as nano-stem, MoS_2_ nanosheets are grown on the surface of MWCNTs/PANI. The amount of the MoS_2_ nanosheets can be controlled by the added amount of ammonium molybdate tetrahydrate. When adding 0.06 g of ammonium molybdate tetrahydrate, only a few sheet-like petal structures of MoS_2_ are formed on the surface of MWCNTs/PANI (shown in figure 2*c* and electronic supplementary material, figure S1*a*). By increasing the amount of ammonium molybdate tetrahydrate to 0.1 g, the MPM-0.1 reveals an exactly same case, in which the individual nanoflower-like MoS_2_ nanosheets are homogeneously and regularly formed on the MWCNTs/PANI nano-stem (figure 2*d*,*f*). The average grain diameter of the spherical MoS_2_ nanoflowers is estimated to be approximately 400 nm. The TEM image of MPM-0.14 with the amount of ammonium molybdate tetrahydrate of 0.14 g indicates that excessive MoS_2_ nanosheets are agglomerated into the bulk and are firmly wrapped on the surface of the MWCNTs/PANI nano-stem (shown in figure 2*e* and electronic supplementary material, figure S1*b*).

**Figure 3.**
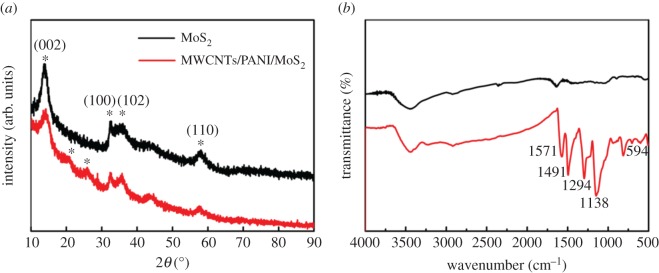
(*a*) XRD patterns and (*b*) FT-IR spectra of MoS_2_ and MWCNTs/PANI/MoS_2_ hybrid.

The crystal structures of the MoS_2_ and MWCNTs/PANI/MoS_2_ nanocomposites are characterized by X-ray diffraction (XRD), as shown in figure 3*a*. For MoS_2_, the characteristic peaks at 2*θ* = 13.74°, 32.63°, 36.21° and 57.84° correspond to (002), (100), (102) and (110), which conforms to the hexagonal structure of MoS_2_. For MWCNTs/PANI/MoS_2_ nanocomposites, all diffraction peaks of MoS_2_ can be observed. Moreover, the nanocomposites also show another two peaks at 20.22° and 25.31°. This is due to the characteristic patterns of PANI, revealing the parallel and perpendicular feature of the polymer chain [36]. Additionally, the Fourier transform infrared (FT-IR) spectra of MoS_2_ and the MWCNTs/PANI/MoS_2_ hybrid are shown in figure 3*b*. For the hybrid, the bands at 1571 cm^−1^ and 1491 cm^−1^ are associated with the C═C stretching vibration of quinonid (Q) and benzenoid (B) rings of PANI. The peaks at 1294 cm^−1^ and 1138 cm^−1^ can be attributed to the C–N stretching vibration of the secondary aromatic amine ring and C–H stretching vibration, in which the C–H band is associated with the conductivity and the degree of electron delocalization of PANI in the nanocomposites [37]. The weak peaks at about 594 cm^−1^ of both MoS_2_ and MWCNTs/PANI/MoS_2_ nanocomposites are attributed to Mo--S vibration [21].

### 3.2. Electrochemical properties

**Figure 4.**
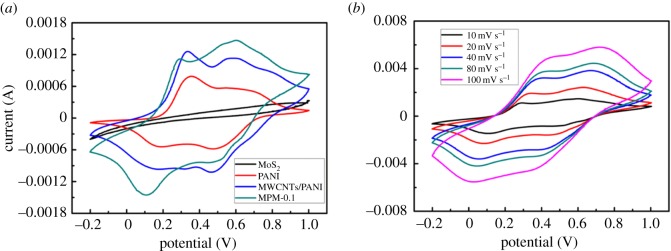
(*a*) CV curves of MoS_2_, PANI, MWCNTs/PANI and MPM-0.1 at 10 mV s^−1^ in 1 M H_2_SO_4_ electrolyte and (*b*) CV curves of MPM-0.1 nanocomposite at different scan rates (10 mV s^−1^, 20 mV s^−1^, 40 mV s^−1^, 80 mV s^−1^, 100 mV s^−1^) in 1 M H_2_SO_4_.

Figure 4*a* illustrates the CV curves comparing MoS_2_, PANI, MWCNTs/PANI and MPM-0.1 at a scan rate of 10 mV s^−1^. It is observed that the CV curve of MoS_2_ is a rectangular shape, revealing that MoS_2_ exhibits typical electric double-layer capacitive character. However, for PANI, MWCNTs/PANI and MPM-0.1, all electrodes exhibit higher current value and two pairs of redox peaks, which are related to the transition of leucoemeraldine/emeraldine and emeraldine/pernigraniline of PANI during the cycling process. Furthermore, the shape of MPM-0.1 is slightly different from that of PANI and MWCNTs/PANI, which is attributed to the introduction of MoS_2_. The CV plots of MPM-0.06 and MPM-0.14 are shown in electronic supplementary material, figure S2. It is clearly seen that the integration area of the CV curve for MPM-0.1 is larger than that for MPM-0.06 and MPM-0.14, indicating a desirable capacitance. Figure 4*b* represents the representative CV curves of the MPM-0.1 nanocomposite at different scan rates. The peak current density and the CV loop area of the hybrid increase clearly with the increase in scanning rates, demonstrating the excellent rate property of the hybrid [38].

**Figure 5.**
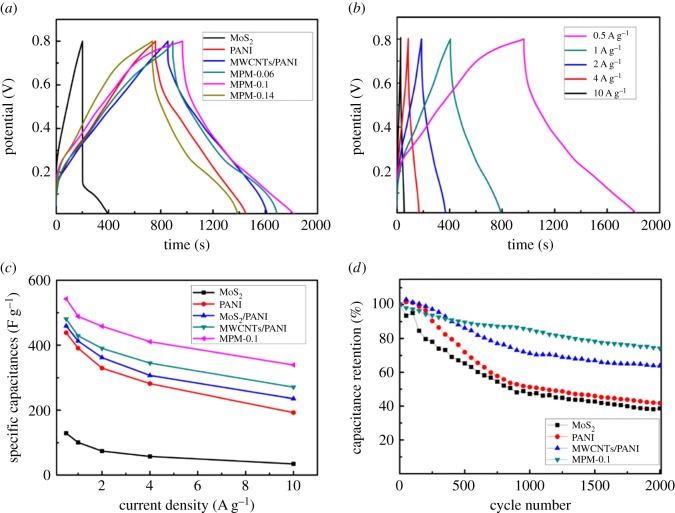
(*a*) GCD plots of MoS_2_, PANI, MWCNTs/PANI, MPM-0.06, MPM-0.1 and MPM-0.14 at a current density of 0.5 A g^−1^ in 1 M H_2_SO_4_; (*b*) GCD plots of MPM-0.1 at different current densities. (*c*) Variation of the specific capacitance with different current density for MoS_2_, PANI, MoS_2_/PANI, MWCNTs/PANI and MPM-0.1 nanocomposites. (*d*) Cycling performance of MoS_2_, PANI, MWCNTs/PANI and MPM-0.1 at 1 A g^−1^ for 2000 cycles.

The representative GCD curves of MoS_2_, PANI, MWCNTs/PANI, MPM-0.06, MPM-0.1 and MPM-0.14 at a current density of 0.5 A g^−1^ are displayed in figure 5*a*. The specific capacitance values of the six electrodes are 128.75 F g^−1^, 438.46 F g^−1^, 480.8 F g^−1^, 498.32 F g^−1^, 542.56 F g^−1^ and 410.27 F g^−1^, respectively, calculated by equation (2.1). It is obvious that the MPM-0.1 electrode possesses larger specific capacitances than other electrode materials. This can be due to the fact that the nanoflower-like MoS_2_ formed on the surface of MWCNTs/PANI produces large double-layer capacitance and this kind of nanostructure does not obstruct the transmission of protons and electrons between the PANI surface and H_2_SO_4_ electrolyte, which can maintain the faradaic pseudocapacitance property of PANI. Furthermore, the excellent electrochemical properties are attributed to the synergistic effect of MWCNTs, PANI and MoS_2_. By contrast, MPM-0.06 possesses a few sheet-like petal structures of MoS_2_, which can provide small double-layer capacitance. MPM-0.14 with a great number of MoS_2_ nanosheets exhibits a smaller specific capacitance. This phenomenon can be due to the excessive MoS_2_ nanosheets agglomerated into the bulk and wrapped on the surface of MWCNTs/PANI, which block electron transfer between PANI and the electrolyte.

Figure 5*b* presents the GCD curves of the MPM-0.1 electrode measured at different current densities. The variation of the specific capacitance values of MoS_2_, PANI, MoS_2_/PANI, MWCNTs/PANI and MPM-0.1 nanocomposite electrodes at different densities is plotted in figure 5*c*. The specific capacitance retention of the MPM-0.1 electrode obtained from the discharge values is 62.5% (from 542.56 F g^−1^ to 339.15 F g^−1^) as the current density increases from 0.5 to 10 A g^−1^, which is higher than those of the MoS_2_ electrode (26.75%), pure PANI electrode (43.9%), MoS_2_/PANI (51.3%) and MWCNTs/PANI electrode (56.35%). The result indicates the MPM-0.1 hybrid electrode possesses a high rate of capacitance, which is recognized as one of the most important electrochemical properties in the application of electrodes. Moreover, comparing the rate performances of MoS_2_/PANI and MPM-0.1, it is found that the existence of MWCNTs could improve the stability of the electrode materials and consequently enhance the discharge performance of supercapacitors.

The evaluation of the cycling stability of the four electrodes is carried out by GCD cycling at a current density of 1 A g^−1^ for 2000 cycles (figure 5*d*). It is found that the specific capacitance retention of the MPM-0.1 hybrid electrode is 74.25%, which is higher than those of MoS_2_ (38.6%), PANI (41.76%) and MWCNTs/PANI (61.82%). Moreover, the cycling performance of MPM-0.1 for 3000 cycles was also tested. Its specific capacitance retention is 73.71% (electronic supplementary material, figure S3), revealing good cycle performance and stability of the hybrid. The improved stability is mainly attributed to the architecture with synergistic effect of nanoflower-like MoS_2_, MWCNTs and PANI. Firstly, MWCNTs acts as a framework to make PANI effectively accommodate the mechanical deformation caused by the swelling and shrinking of the nanostructures during the long-term GCD process. Secondly, the nanoflower-like MoS_2_ covering on the surface of MWCNTs/PANI can suppress the volume change of PANI from the outside, which avoids the destruction of the electrode material and leads to outstanding stability.

## 4. Conclusion

In summary, a novel structured hybrid with nanoflower-like MoS_2_ suitably grown on the surface of MWCNTs/PANI is successfully prepared by a facile *in situ* polymerization and hydrothermal method. In the hybrid, nanoflower-like MoS_2_ not only acts as an active material with double-layer capacitance but also as an outer barrier to suppress the volume change of PANI; MWCNTs serve as a support framework to make PANI effectively accommodate the mechanical deformation and to improve the electrochemical property of the whole material; PANI provides the faradaic contribution to the overall capacitance. Electrochemical measurements show that the MWCNTs/PANI/MoS_2_ hybrid electrode displays an ideal specific capacitance of 542.56 F g^−1^ at a current density of 0.5 A g^−1^ and excellent cycling stability with a capacitance retention of 73.71% after 3000 cycles, indicating its potential for high-performance electrical energy storage.

## Supplementary Material

Highlights;Prime Novelty Statement

## Data accessibility

The datasets supporting this article have been uploaded as part of the electronic supplementary material.
