# Supplementary material for: Novel ternary nanocomposites of MWCNTs/PANI/MoS2: preparation, characterization and enhanced electrochemical capacitance
Source: R Soc Open Sci. 2018 Jan 3;5(1):171365. doi: 10.1098/rsos.171365 (PMC5792917; doi:10.1098/rsos.171365)
Supplement: Highlights;Prime Novelty Statement [file rsos171365supp1.docx]

**Novel** **ternary nanocomposites of MWCNTs/PANI/MoS_2_: preparation, characterization and enhanced electrochemical capacitance**

**Ranran Zhang‡, Yu Liao‡, Shuangli Ye, Ziqiang Zhu, Jun Qian**


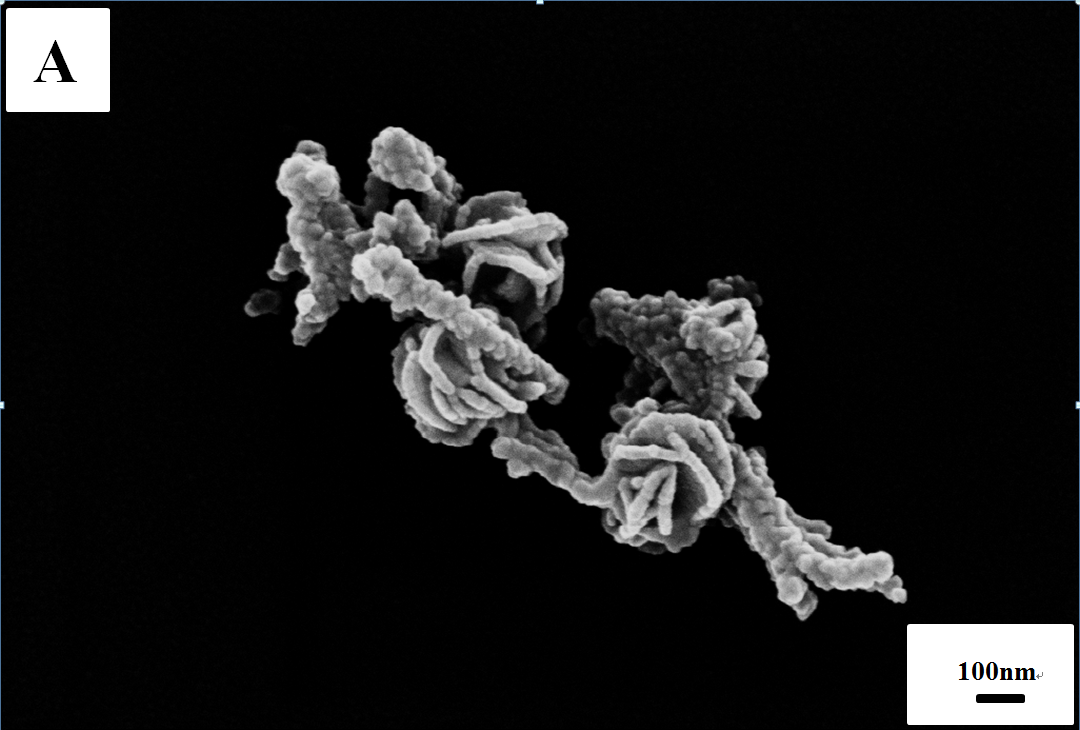

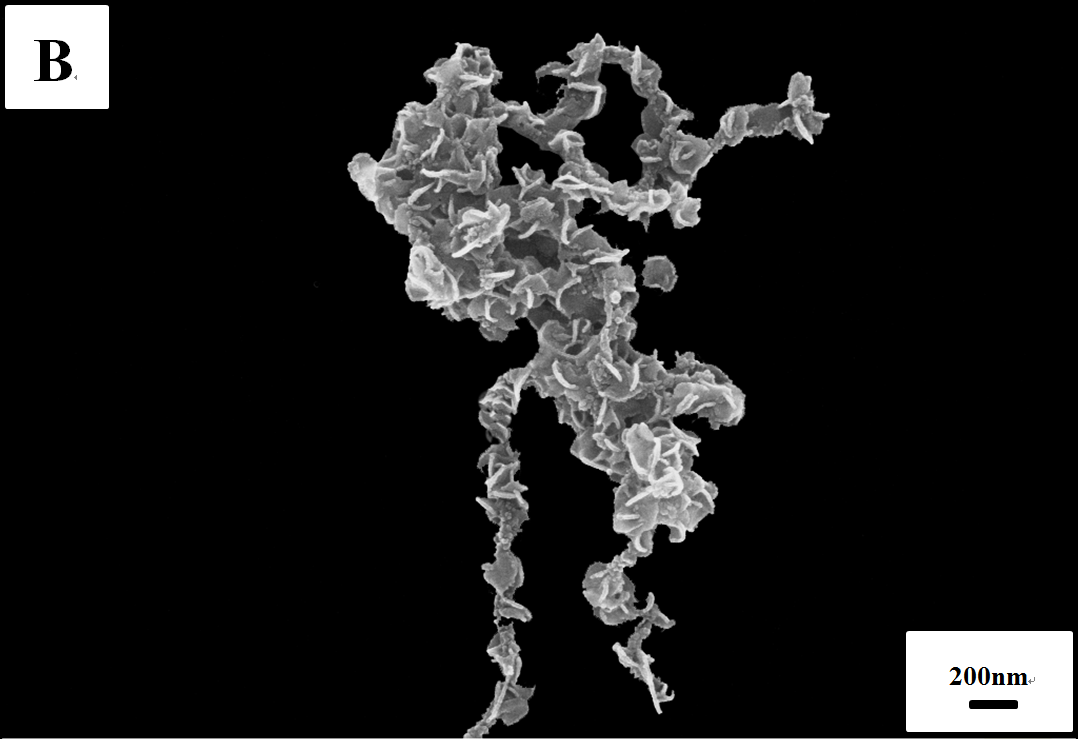


**Figure.**S1 SEM images of MPM-0.06 (A) and MPM-0.14 (B)





**Figure.**S2 CV curves of MPM-0.06, MPM-0.1 and MPM-0.14 at 10 mVs^-1^ in 1M H_2_SO_4_ electrolyte

**
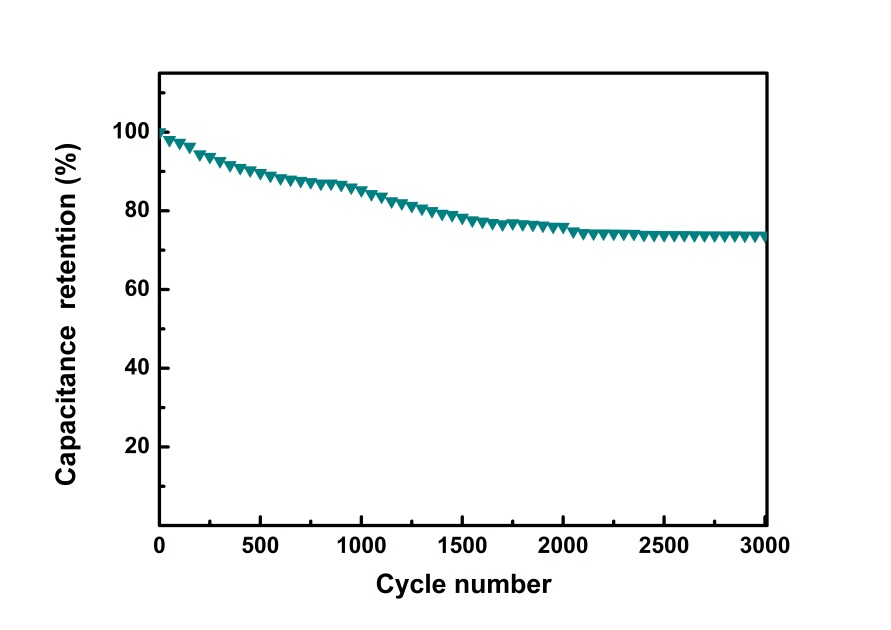
**

**Figure.**S3 The cycling performance of MPM-0.1 at 1 A g^-1^ for 3000 cycles
